# Truncated and Helix-Constrained Peptides with High Affinity and Specificity for the cFos Coiled-Coil of AP-1

**DOI:** 10.1371/journal.pone.0059415

**Published:** 2013-03-27

**Authors:** Tara Rao, Gloria Ruiz-Gómez, Timothy A. Hill, Huy N. Hoang, David P. Fairlie, Jody M. Mason

**Affiliations:** 1 School of Biological Sciences, University of Essex, Colchester, United Kingdom; 2 Division of Chemistry and Structural Biology, Institute for Molecular Bioscience, The University of Queensland, Brisbane, Queensland, Australia; Universidad de Granada, Spain

## Abstract

Protein-based therapeutics feature large interacting surfaces. Protein folding endows structural stability to localised surface epitopes, imparting high affinity and target specificity upon interactions with binding partners. However, short synthetic peptides with sequences corresponding to such protein epitopes are unstructured in water and promiscuously bind to proteins with low affinity and specificity. Here we combine structural stability and target specificity of proteins, with low cost and rapid synthesis of small molecules, towards meeting the significant challenge of binding coiled coil proteins in transcriptional regulation. By iteratively truncating a Jun-based peptide from 37 to 22 residues, strategically incorporating i→i+4 helix-inducing constraints, and positioning unnatural amino acids, we have produced short, water-stable, α-helical peptides that bind cFos. A three-dimensional NMR-derived structure for one peptide (24) confirmed a highly stable α-helix which was resistant to proteolytic degradation in serum. These short structured peptides are entropically pre-organized for binding with high affinity and specificity to cFos, a key component of the oncogenic transcriptional regulator Activator Protein-1 (AP-1). They competitively antagonized the cJun–cFos coiled-coil interaction. Truncating a Jun-based peptide from 37 to 22 residues decreased the binding enthalpy for cJun by ∼9 kcal/mol, but this was compensated by increased conformational entropy (TΔS ≤7.5 kcal/mol). This study demonstrates that rational design of short peptides constrained by α-helical cyclic pentapeptide modules is able to retain parental high helicity, as well as high affinity and specificity for cFos. These are important steps towards small antagonists of the cJun-cFos interaction that mediates gene transcription in cancer and inflammatory diseases.

## Introduction

Cellular functions are mediated by protein-protein interactions, the majority involving large interacting surface areas with binding interfaces that are shallow, hydrophilic and lack the well-defined small hydrophobic clefts that are most tractable for design of small molecule inhibitors. Driven by the need to target these larger protein surfaces, there has been renewed interest in recent years in developing larger therapeutic molecules, such as peptides and their mimetics, which can in principle combine advantages of proteins (target specificity, structural stability) with advantages of small molecules (lower cost, oral activity) [1]. One approach is to engineer small synthetic components of protein surfaces (‘protein surface mimetics’) [2] to compete for (antagonists) or mimic (agonists) protein-protein interactions that mediate disease. However, peptides have also traditionally been perceived as being problematic therapeutics as they are often considered too large, too polar and too susceptible to protease degradation in order to traverse intact across biological membranes. Conjugating small (5–12 residue) protein-transduction domains or arginine-rich peptides, such as TAT and antennapedia fragments [3], [4], [5] to peptide cargo can be used to facilitate cell penetration. However, those sequences increase peptide size and are themselves susceptible to proteolytic degradation. Rendering peptides protease resistant has been more difficult to engineer without replacing key components with non-peptidic groups, or creating N- to C-terminal ‘cyclic peptides’, to bring stability and bioavailability to the peptide (see [1], [2], [3] and references therein). More recently, constraints have been incorporated into peptide sequences to induce bioactive helix, strand or turn structural motifs that have high affinity for receptors without the need for larger sequences [6], [7], [8], [9], [10], [11]. Alpha helices have been successfully stabilized by introducing constraints in the side-chains of amino acids [12], [13], [14], [15], [16], [17], [18], [19] or within the peptide backbone using hydrogen bond surrogate approaches [20], [21], [22], [23], [24], [25], [26], [27], [28], [29], [30]. In the HBS approach α-helices feature a carbon−carbon bond in place of an N-terminal intramolecular hydrogen bond between the peptides i and i +4 residues. Thus organizing three consecutive amino acids into the helical orientation inherently limits the stability of short α-helices. The HBS method affords preorganized α-turns to overcome this intrinsic nucleation barrier and initiate helix formation.Other approaches include β-peptides [31], [32], without interfering with the helix surface designed to interact with the target protein, thereby conferring high helicity that confers protein-like function upon peptides that would otherwise have low or negligible biological potency. We have previously used one-turn (i→i+4) rather than two-turn (i→i+7) bridging constraints to induce α-helicity [9], [11], [33], [34], since our research supports greater per residue helicity even though this is contrary to polymer theory [35]. The approach can however be context dependent, and requires extensive further investigation to realise its promise.

**Figure 1:**
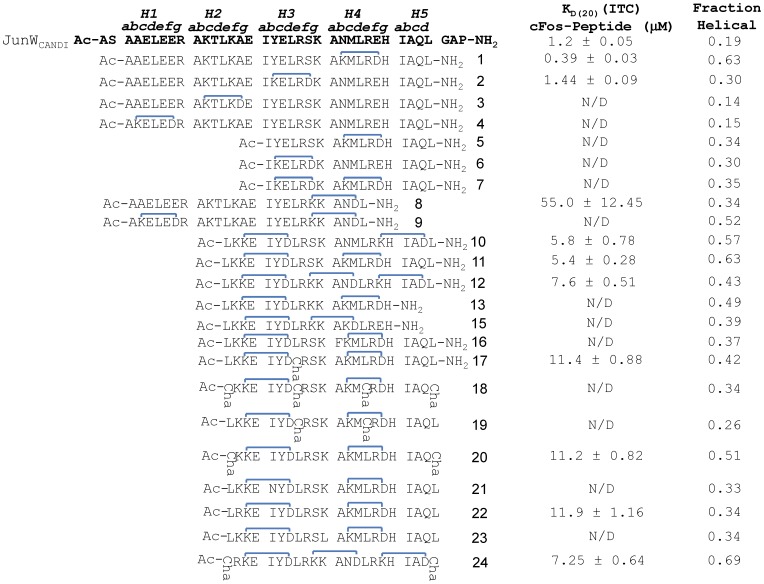
Schematic showing sequences and constrains for all peptides. The parental JunW_CANDI_ sequence is shown in bold as are heptads and residue positioning within the helical wheels. Peptide constraints are shown in blue. K_D_ values taken from ITC experiments for peptides in complex with cFos are shown in µM. Fraction helicity as measured from CD experiments are also shown. Positions of *i*→*i+4* hydrocarbon constraints were initially placed into a JunW_CANDI_ peptide [42] lacking capping motifs, causing a reduction in the size of the molecule from 37 residues to 32. All constraints tethered *b*→*f* or *f*→*c* residues.

The Jun-Fos Activator Protein-1 (AP-1), is a helical heterodimer and oncogenic transcriptional regulator implicated in a range of diseases that includes cancer [36], [37], [38], bone disease (e.g. osteoporosis) and inflammatory diseases such as rheumatoid arthritis and psoriasis [39], [40], [41]. A number of intracellular signalling cascades converge at AP-1, producing changes in gene expression profiles that can cause tumour formation, progression and metastasis. Here we begin with a 37 residue peptide (JunW_CANDI_) found to bind specifically to cFos in the presence of cJun [42] by binding to the coiled coil region that is responsible for driving AP-1 heterodimerization. In brief, the coiled coil is characterised by a repeat of seven amino acids, denoted ***a-g***; residues ***a*** and ***d*** consist largely of hydrophobic residues, forming a stripe which associates with respective partners on the other helix. Core flanking charged residues at ***e*** and ***g*** positions form interhelical ion pairs with ***g*** and ***e***_residues in the neighbouring helix and aid heterospecificity [43]. Core region proximity means these residues are also partially shielded from the solvent [44]. Since the Jun-Fos dimer interface is responsible for mediating key transcriptional events associated with disease induction, it may be a worthy target for therapeutic intervention. We have previously reported peptides of 33–37 amino acids, based on the coiled coil region known to control dimerization [45], [46], and flanked by N- and C-terminal capping motifs, that were able to bind and sequester either cJun or cFos to prevent Jun-Fos heterodimer formation, initiation of gene transcription, and cell differentiation and proliferation [42], [47], [48], [49]. These peptides were however too large to be useful therapeutics. By systematically truncating the 37 residue peptide ‘JunW_CANDI_’ [42], together with strategic introduction of helix constraints (Figure 1), we sought to establish (i) whether helix-constrained peptides could be significantly reduced in length while maintaining effective binding; (ii) whether the downsized peptides maintained high binding specificity for cFos relative to cJun; (iii) whether there was a key consensus region within the coiled coil that was essential for binding; and (iv) whether the approach required mainly enthalpic or entropic contributions to be successful. The latter is important because coiled coils typically require interactions along their entire lengths for structural stability.

We report that α-helical cyclic pentapeptide modules inserted into truncated sequences from within the JunW_CANDI_ peptide results in much shorter water-stable α-helical peptides that retain the high affinity and specificity of the parental JunW_CANDI_ peptide for cFos, and are stable to proteolytic degradation. Affinity for cFos is driven by a combination of interactions along most of the sequence of cJun, and we were able to pinpoint key co-facial residues that contribute to the overriding enthalpic properties that dictate peptide potency. This is an important step forward in understanding how to rationally design small transcriptional regulators.

## Experimental Procedures

### Peptide Synthesis and Purification

Peptide synthesis was performed as described [9], [11], [33] by Fmoc chemistry. The phenyl isopropyl ester of aspartic acid and methyl trityl group of lysine were removed from the peptide-resin with 3% TFA in dichloromethane (DCM) (5×2 min). Cyclization was effected on-resin using Benzotriazole-1-yl-oxytris-(dimethylamino)-phosphonium hexafluorophosphate (BOP) and 1-hydroxy-7-aza-benzotriazole (HOAt), base N,N-Diisopropylethyamine (DIPEA), and DMF (1⋮1). The procedure was repeated for multiple cyclizations. Crude peptides were purified by rp-HPLC (Rt1: Vydac C18 column, 300 Å. 22×250 mm, 214 nm, Solvent A = 0.1% TFA in H2O, Solvent B = 0.1% TFA, 10% H_2_O in acetonitrile. Gradient: 0% B to 70% B over 35 min). Peptides were >95% purity by analytical HPLC. Correct masses were verified by electrospray mass spectrometry. Peptide masses were as follows: cFos = 4147; ***1*** = 3747; ***2*** = 3740; ***3*** = 3792; ***4*** = 3791; ***5*** = 2208; ***6*** = 2201; ***7*** = 2183; ***8*** = 2926; ***9*** = 2951; ***10*** = 2661; ***11*** = 2675; ***12*** = 2668; ***13*** = 2291; ***15*** = 2287; ***16*** = 2751; ***17*** = 2701; ***18*** = 2786; ***19*** = 2730; ***20*** = 2730; ***21*** = 2675; ***22*** = 2704; ***23*** = 2661; ***24*** = 2751. All synthetic peptides were N- acetylated and C-amidated. Peptide concentrations were determined by dry weight and verified via absorbance in water at 280 nm with an extinction coefficient of 1209 M^−1^ cm^−1^ [50] corresponding to a single Tyr residue inserted into a solvent-exposed ***b3*** heptad position. The peptide concentration for ***2, 6*** and ***7*** were determined by dry weight alone since the ***b3*** Tyr was replaced by an Lys residue that formed part of the helix constrained peptide.

### NMR Spectroscopy

**Figure 2:**
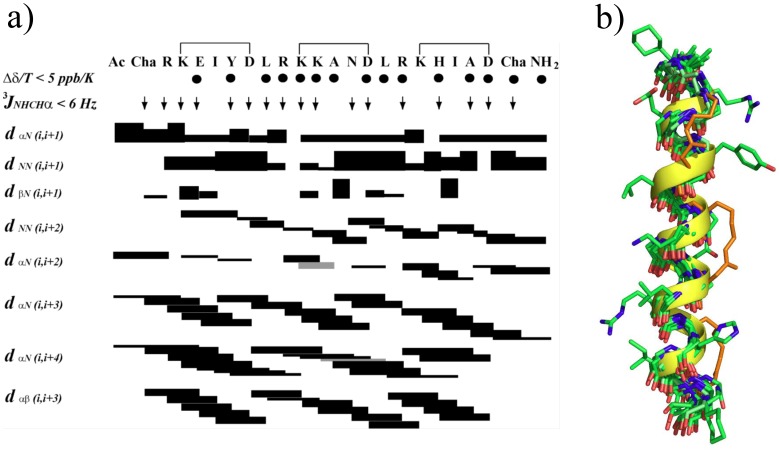
NMR Structure of peptide 24. **a)** NOE summary diagram for peptide **24** in 90% H_2_O:10% D_2_O at 298 K. Sequential, short and medium range NOE intensities were classified as strong (upper distance constraint 2.7 Å), medium (3.5 Å), weak (5.0 Å), very weak (6.0 Å) and are proportional to bar thickness; grey bars indicate overlapping signals. ^3^ *J*_NHCHα_coupling constants <6 Hz are indicated by ↓. Amide NH’s for which chemical shifts changed by <5 ppb/K are indicated by •. **b)** Backbone superimposition for ten lowest energy NMR-derived solution structures for Ac-cyclo-(3,7; 10,14; 17,21)-ChaR[KEIYD]LR[KKAND]LR[KHIAD]Cha-NH_2_ (**24**) in H_2_O:D_2_O (9∶1) at 298 K showing carbon atoms (green), nitrogens (blue), oxygens (red), *i*→*i+4* hydrocarbon constraints (orange). Also for clarity, one structure is shown with its alpha helical backbone (yellow) and projecting side chains (green). N-terminus is at the top.

A sample for NMR analysis (Figure 2) was prepared by dissolving peptide **24** (2.0 mg) in 540 µL H_2_O and 60 µL D_2_O. 1D (variable temperature experiments) and 2D ^1^H-NMR spectra were recorded on a Bruker Avance 600 and 900 MHz spectrometers respectively. 2D ^1^H-spectra were recorded in phase-sensitive mode using time-proportional phase incrementation for quadrature detection in the *t*1 dimension. 2D experiments included TOCSY (standard Bruker mlevgpph pulse program) and NOESY (standard Bruker noesygpph pulse program) and *dqf*-COSY (standard Bruker dqfcosygpph pulse program). TOCSY spectra were acquired over 9920 Hz with 4096 complex data points in *F_2_*, 256 increments in *F_1_* and 32 scans per increment. NOESY spectra were acquired over 9920 Hz with 4096 complex data points in *F_2_*, 512 increments in *F_1_* and 32 scans per increment. TOCSY and NOESY spectra were acquired with several isotropic mixing times of 80 ms for TOCSY and 200–250 ms for NOESY. For all water suppression was achieved using modified WATERGATE and excitation sculpting sequences. For 1D ^1^H NMR spectra acquired in H_2_O/D_2_O (9∶1), the water resonance was suppressed by low power irradiation during the relaxation delay (1.5 to 3.0 s). Spectra were processed using Topspin (Bruker, Germany) software and NOE intensities were collected manually. The *t*1 dimensions of all 2D spectra were zero-filled to 1024 real data points with 90° phase-shifted QSINE bell window functions applied in both dimensions followed by Fourier transformation and fifth order polynomial baseline correction. Variable temperature NMR experiments were performed over 278–318 K. ^1^H chemical shifts were referenced to DSS (δ 0.00 ppm) in water. ^3^ *J*_NHCHα_ coupling constants were measured from 1D ^1^H NMR and *dqf*-COSY spectra. The assigned ^1^H NMR signals for peptide 24 can be found in Table S2.

### Structure Calculations

The distance restraints used in calculating a solution structure for ***24*** in water was derived from NOESY spectra recorded at 298 K or 288 K by using mixing time of 250 ms. NOE cross-peak volumes were classified manually as strong (upper distance constraint ≤ 2.7 Å), medium (≤ 3.5 Å), weak (≤ 5.0 Å) and very weak (≤ 6.0 Å) and standard pseudo-atom distance corrections were applied for non-stereospecifically assigned protons. To address the possibility of conformational averaging, intensities were classified conservatively and only upper distance limits were included in the calculations to allow the largest possible number of conformers to fit the experimental data. Backbone dihedral angle restraints were inferred from ^3^ *J*_NHCHα_ coupling constants in 1D spectra, ϕ was restrained to –60±30° for ^3^ *J*_NHCHα_ ≤ 6 Hz. Starting structures with randomized ϕ and ψ angles and extended side chains were generated using an *ab initio* simulated annealing protocol. The calculations were performed using the standard force field parameter set (PARALLHDG5.2.PRO) and topology file (TOPALLHDG5.2.PRO) in XPLOR-NIH with in house modifications to generated i→i+4 helix constraints between lysine and aspartic acid residues and unnatural amino acid Cyclohexylalanine (Cha). Refinement of structures was achieved using the conjugate gradient Powell algorithm with 2000 cycles of energy minimization and a refined force field based on the program CHARMm [51]. Structures were visualized with Pymol and analyzed for distance (>0.2 Å) and dihedral angle (>5°) violations using noe.inp and noe2emin.inp files (in Xplor). Final structures contained no distance violations (>0.2 Å) or angle violations (>5°). Corresponding NMR coordinates are available upon request.

### Serum Stability

**Figure 3:**
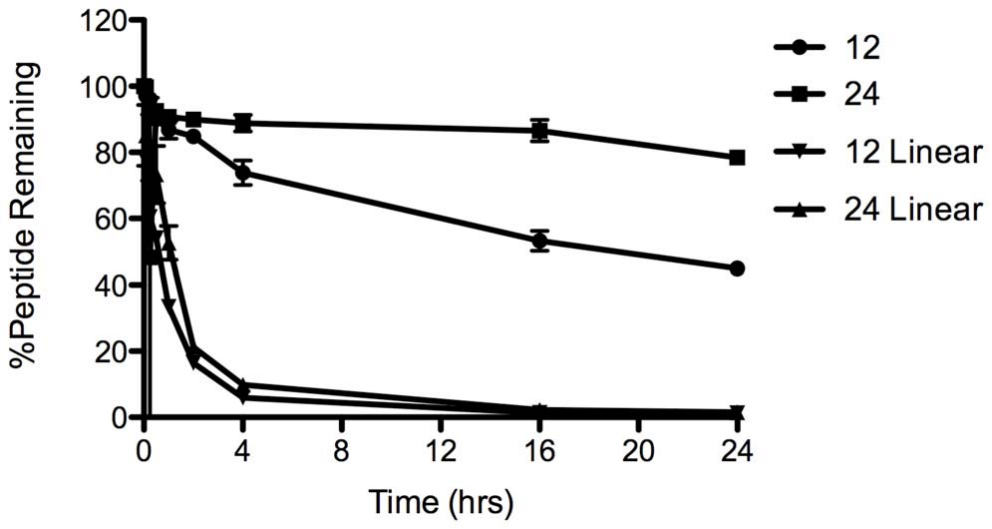
Serum stability of peptides 12 and 24. Shown are the effects of helix-inducing constraints (• and ▪) versus the linear sequences (▾ and ▴) in human serum at 37°C.

Stock solutions of ***12*** and ***24*** in both constrained forms and linear forms lacking constraints were prepared in water (1 mg/ml), 200 µL was added to human serum (800 µL) and incubated at 37°C. Aliquots (100 µL) of this diluted serum were removed at 0, 0.5, 1, 2, 4, 16, and 24 hours and a mixture of acetonitrile/water 3∶1 (300 µL) was added to each aliquot before centrifuging (17000 rpm, 15 min). Aliquots (100 µL) of the supernatant were then analysed by LC-MS-MS after passing through a 2.1×150 mm Phenomenex 300A C18 5 µm column at 10% per minute linear gradient from 0–100% acetonitrile over 12 minutes. The amount of starting material was quantified by determination of total ion counts for the m/3+ and m/4+ ion for each peptide (Figure 3).

### Circular Dichroism

**Figure 4:**
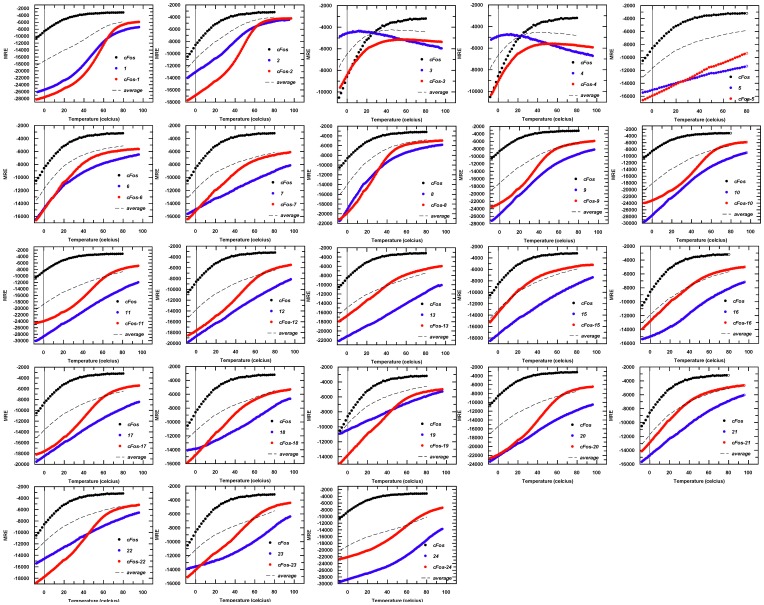
Raw thermal melting data for all homo and heterodimeric complexes. Data have been collected by measuring the level of helicity at 222 nm in an applied photophysics chirascan Circular Dichroism (CD) Spectrometer. Data have been converted from raw ellipticity to Molar Residue Ellipticity (MRE) according to equation 1 to take account of the different peptide lengths. Thermal melting data for cFos is shown in black, data for the constrained peptide in isolation is shown in blue and the cFos/constrained peptide mixture is shown in red. Also shown is the average of the cFos and constrained peptide (black dotted line). Where possible data have been fitted to equation 2 to generate thermal melting (T_m_) values (e.g. for cFos-***1*** and cFos-***2***) and in such instances it is clear from an increase in the averaged homomeric T_m_ values that an interaction is occurring (e.g. ***1***: −1+50 = 49/2 = 24.5<55). However, some data were unable to be fitted owing to the lack of a melting transition, or of a lower baseline (e.g. cFos-***3*** and cFos-***4***), indicating that an interaction is not occurring. CD spectra for these pairs are given in Figure S3.

Circular dichroism (CD) spectroscopy was conducted with an Applied Photophysics Chirascan CD spectroploarimeter (Leatherhead, U.K.) using a 200 µL sample in a CD cell with a 1 mm path length (Hellma, Müllheim Germany). Samples contained 150 µM total peptide concentration suspended in 10 mM potassium phosphate and 100 mM potassium fluoride at pH 7. The CD spectra of the homodimeric and heterodimeric complexes were scanned between 300 nm and 190 nm at 20°C (for both pre- and post-melt samples to check for reversibility of unfolding) and at −8°C to assess helical levels and coiled-coil structure (see Figure 4). All data have been converted from raw ellipticity to molar residue ellipticity according to the equation:
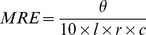
(1)Where θ is the CD signal of the sample in millidegrees, l is the pathlength of the cell in centimetres, r is the number of residues in the peptide, and c is the total peptide molar concentration of the sample.

### Thermal Melts

Spectra and thermal melts were performed on 150 µM peptides in 10 mM potassium phosphate, 100 mM potassium fluoride, pH 7, using an Applied Photophysics Chirascan CD instrument (Leatherhead, U.K.). The temperature ramp was set to stepping mode using 1°C increments and paused for 30 seconds at each temperature before measuring ellipticity at 222 nm. For all temperature denaturation experiments data collection was started at −8°C, and at this temperature the peptide solutions remained aqueous. Data points for thermal denaturation profiles represent the averaged signal after 4 s of data collection. Samples were identical in composition to the CD buffer samples. Melting profiles (see Figure 4) were ≥95% reversible with equilibrium denaturation curves fitted to a two-state model (see [52]) to yield the melting temperature (T_m_):
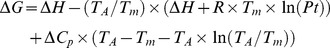
(2)where ΔH is the change in enthalpy, T_A_ is the reference temperature in Kelvin; R is the ideal gas constant (1.9872 cal⋅mol^–1^⋅K^–1^); P_t_ the total peptide concentration (150 µM); and ΔC_p_ the change in heat capacity [47]. Helix heterodimerisation is inferred in cases where melting profiles for heterodimers are clearly distinct from averages of constituent homodimeric melts (Shown in Figure 4 and via dimer exchange in Figure S3). The cooperative nature of the melting profiles suggests an apparent two-state process. T_m_ values were determined by least-squares fitting of the denaturation assuming a two-state folding model that is widely used for coiled coils and provided an excellent fit to our data.

### Inspection of Homo and Heterotypic CD Data

Thermal denaturation data and spectra for the cFos homotypic complex (black), helix constrained peptide (blue), and heteromeric complex (red) were recorded using 200 µL of sample at 150µM total peptide concentration (Figure 4, Figure S1). Next, 100 µL of each solution was mixed and the spectra taken such that the final total peptide concentration was also 150 µM. More experimental procedures can be found in File S1.

## Results

### Design Rational and Evolution of the Helix-constrained Peptide Sequences

Our first aim was to systematically introduce helix-inducing constraints into JunW_CANDI_ which has been shown to be specific for cFos in the presence of cJun [42]. All helix-constrained peptides lack five residues that served as N-terminal and C-terminal capping motifs in the JunW_CANDI_ parent peptide [42], [47], [48]. This led us to examine the effect on helix induction of one or more such constraints placed at different positions within the sequence, while concomitantly truncating the sequence from either terminus. The goal was to decrease the peptide length while maintaining the peptide helicity using one or more helix-inducing constraints. The process was iterative, generating 24 peptides. We find that introduction of helical constraints leads to a stable heterodimeric peptide with cFos for some but not all of the constructs developed, indicating that induction of helicity alone is insufficient to achieve binding:

### Peptides 1–4

Our initial series of four constructs (Figure 1) were based on the JunW_CANDI_ scaffold (4.5 heptads in length), but lacked the N- and C-terminal capping motifs. Thus at 32 residues these peptides were five residues shorter than this *‘Protein-fragment complementation assay’* (PCA) derived parent peptide [42], [47], [48], but retained all regions of the coiled coil. The first four peptides synthesized (**1–4**, Figure 1) each had one i→i+4 helix constraint formed by inserting Lys and Asp in place of solvent exposed residues between positions ***b*** and ***f*** in one of the four heptads of the coiled coil. Circular dichroism spectra were used to measure the relative extent of α-helix induction and showed that we were successful in our design process (Table S1, Figure S1). Constraints in either of the two C-terminal heptads were more effective than in the two N-terminal heptads at inducing α-helicity (**1**, 63%; **2**, 30%; **3**, 14%; **4**, 15%; Table S1), suggesting an important effect of the sequence environment surrounding the constraint which was most helix-inducing when placed in the most C-terminal heptad (e.g. **1**). This led to two peptides that bound (***1*** and ***2***– constrained at the C-terminal end of the molecule; see Figure 1) and two that did not (***3*** and ***4***– constrained at the N-terminal end). Our interpretation of this result was that the first two heptads are less important in binding cFos and that the latter two heptads were crucial. Indeed, cFos-***1*** displays a T_m_ of 55°C; 11°C higher than the cFos-JunW_CANDI_ [42], while cFos-***2*** displays a T_m_ of 53°C, amounting to K_D_ values of approximately 1 µM as verified by ITC (see Table 1). In contrast, cFos-***3*** and cFos-***4*** did not generate a thermal denaturation profile that could be fitted to equation 2; rather the profile lacked a lower baseline and entered partway through the unfolding transition (see Figure 4). The data also shows that ***3*** or ***4*** in solution with cFos results in much lower helicity than cFos with ***1*** or ***2*** (cFos-***1,*** 65%; cFos-***2,*** 39%), compared to ***3*** or ***4*** (cFos-***3***, 17%; cFos-***4***, 26%). For this reason, thermal denaturation data could only be fitted and normalised to a fraction unfolded where satisfactory denaturation profiles, lower baselines and overall fit to equation 2 could be attained. We interpreted cFos-***1*** stability as being attributable to helical propensity resulting from a constraint at the C-terminus, where less helical structure in the parent protein might be assumed. The same outcome is not observed for the N-terminal constrained peptides ***3*** and ***4***, which are significantly less helical. We therefore inferred that the N-terminal region does not play a substantial role in binding, or that these constraints cause steric interference at this end of the molecule. All of these peptides retained ten core ***a/d*** residues per helix for hydrophobic interactions and six electrostatic ***e/g*** interactions (Figure 1).

**Table 1:** Thermodynamics of binding of cJun analogues to cFos. Columns (from left to right) show i) Tm values from thermal denaturation analysis ii) calculated % helicity for each respective pair calculated from circular dichroism spectra and iii) K_D_ values calculated from thermal denaturation data.

Peptide complex	T_m_ °C	% Helical	K_D(20)_ (Thermal) µM	*K_D(20)_ M^−1^ (ITC) µM	N	ΔG_(20)_ kcal/mol (ITC)	ΔH kcal/mol	#TΔS kcal/mol [ΔH – ΔG]
**cFos-cFos**	−1	20%	325	N/D	N/D	N/D	N/D	N/D
**cFos-JunW_CANDI_**	44	42%	0.45	1.2±0.05	0.8±0.01	−7.9±0.03	−14.8±0.3	−6.9±0.3
**cFos-1**	55	65%	0.50	0.39±0.03	0.8±0.01	−8.6±0.04	−11.6±0.1	−3.0±0.1
**cFos-2**	53	39%	0.19	1.44±0.09	1.2±0.01	−7.8±0.04	−7.1±0.1	+0.7±0.1
**cFos-8**	32	38%	21.5	55±12.45	1.0±0.35	−5.7±0.13	−9.5±0.4	−3.8±0.4
**cFos-9**	38	47%	9.2	N/A	N/A	N/A	N/A	N/A
**cFos-10**	49	52%	1.7	5.8±0.78	0.8±0.01	−7.0±0.08	−8.4±0.9	−1.4±0.9
**cFos-11**	47	55%	5.8	5.4±0.28	0.7±0.01	−7.0±0.03	−7.7±0.2	−0.7±0.2
**cFos-12**	52	40%	1.2	7.6±0.51	1.0±0.02	−6.8±0.04	−6.1±0.2	0.7±0.2
**cFos-17**	44	40%	7.8	11.4±0.88	1.0±0.03	−6.6±.0.05	−5.4±0.3	1.2±0.3
**cFos-20**	48	49%	3.8	11.2±0.82	1.1±0.03	−6.6±0.04	−7.5±0.3	−0.9±0.3
**cFos-22**	51	40%	1.4	11.9±1.16	1.4±0.04	−6.6±0.06	−5.0±0.2	1.6±0.2
**cFos-24**	58	52%	7.2	7.25±0.64	1.1±0.03	−6.9±0.05	−8.8±0.4	−1.9±0.4

The remaining three columns give stoichiometry of binding and thermodynamic data calculated from ITC, with TΔS calculated according to the Gibbs Helmholtz equation.

* data calculated using the midpoint of the transition from thermal denaturation profiles (and fit as temperature as a function of lnK_D_, with the fit lnK_D_ = aT+C where a is the gradient, T is the temperature in Celsius and C is the intercept) and calculated at 20°C.

# Calculated according to TΔS = ΔH−ΔG.

### Peptides 5–7

Using data from the first four peptides, our next group of synthetic constrained peptides were synthesized. These were truncated by two heptads at the N-terminus of the peptide (NΔ14, 18 residues) and a single (***5*** and ***6***) or double (bicyclic –***7***) constraint introduced. Much shorter peptides **5** and **6** had a single helix constraint but were not much more helical (34% and 30%) than the expected statistical ratio of just making 5 of the 18 residues (27%) helical due to their presence in the cyclic pentapeptide component, while there was no additive effect of combining both helix-inducing constraints within the same sequence (e.g. as for peptide ***8*** **)**. Unfortunately, none of these peptides resulted in a stable interaction with cFos. Again, no lower baseline for thermal denaturation profiles was observed and therefore no fit could be given in estimation of the T_m_ (see Figure 4 for raw data). The MRE at θ_222_ was approximately −12000 at 20°C, indicating a comparatively low level of helicity ([26–29% helical], Table S1) compared to cFos-***1*** or cFos-***2***. In addition, peptides ***5–7*** were all more helical in isolation than as heteromeric mixtures with cFos, with all transitions unable to produce a thermal denaturation profile indicative of a stable interaction. Peptides ***5–7*** had six core residues per helix for hydrophobic interaction (four less than peptides ***1–4***) and only three electrostatic ***e/g*** interactions (three less than peptides ***1–4***), which may account for the loss in activity.

### Peptides 8–11

Data taken from peptides ***1–7*** was used in the design of four further peptides ***8–11*** (Figure 1). In this group, ***8*** contained the same sequence as ***1*** but with an additional seven residues truncated from the C-terminus (CΔ7, 25 residues). The constraint also resides closer to the C-terminus of the molecule relative to ***1***. The bi-cyclic ***9*** contained the same sequence and constraint as ***8*** but with the additional N-terminal constraint found in the unsuccessful peptide ***4***; its insertion resulted in a modest helicity gain over ***8*** (θ_222_ at 20°C = −21000 *versus.* −14000). The logic behind this was that by constraining both the N-and C-terminus of the peptide, helicity would be propagated and maintained across the entire molecule. Interestingly, cFos-***8*** (which contains one *f*→*c* constraint, 32°C) performed comparably to cFos-***9*** (one N-terminal *b*→*f* constraint and one C-terminal *f*→*c* constraint, T_m_ 38°C), indicating that the inclusion of the N-terminal constraint in this bicyclic peptide had only a modest influence on the interaction stability. Taken together with data for ***4***, this reinforces the argument that inclusion/constraint of this N-terminal region is less important for interaction with cFos. The heterodimeric stability for these two peptides was almost 20°C less than for cFos-***1*** or cFos-***2***, therefore indicating that at least part of the CΔ7 deletion is an important binding determinant. Given the low affinity of the N-terminally truncated constrained peptides ***5–7*** for cFos, and the modest binding affinity afforded by peptides ***8–9***, two bi-cyclic peptides ***10*** and ***11*** were created to make less severe N-terminal truncations than peptides ***5–7*** (NΔ10 *versus* NΔ14, see Figure 1). Importantly, this more modest truncation incorporated the hydrophobic ***‘d’*** residue of the second heptad with the constraint inserted in close proximity, ensuring that the helical integrity of this region was maintained entirely to the helical termini. In addition, ***10*** and ***11*** retained the complete C-terminal region with a second constraint either close to (***11***) or at (***10***) the C-terminus. ***10*** contains an *f2*→*c3* and an *f4*→*c5* constraint whereas peptide ***11*** contains an *f2*→*c3* and a *b4*→*f4* constraint. These peptides increased the T_m_ with cFos by 9–17°C relative to peptides 8–9, and within 4–8°C of peptides ***1*** and ***2*** (and with comparable levels of helicity) despite being 10 residues shorter. Reassuringly, peptides ***8–11*** all give thermal denaturation profiles that can be fit by equation 2. It is also worth noting that although the position of the constraint is always between *b*→*f* or *f*→*c* (and therefore always tethers *i*→*i+4* and is positioned away from the ***a/d/e/g*** residues associated with the dimeric coiled coil interface), it cannot be ruled out that the precise position of the constraint, and the residues that become replaced by the Lys-Asp pair, do not affect binding affinity in the resulting peptide. In addition, although some peptides are very helical in isolation (as would be expected for such conformationally restricted chains), others exhibit no unfolding transition upon thermal melting, with interaction clearly observed via a cooperative unfolding transition upon mixing with cFos. Lastly, peptides ***8–11*** had eight (for ***8–9***) or seven (for ***10–11***) core residues per helix for hydrophobic interaction (two or three respectively less than for ***1–4***) and five electrostatic ***e/g*** interactions (one less than ***1–4***). Despite the fact that more core residues are present in ***8–9***, more stability is observed for cFos in complex with ***10–11***, indicating that reduced core packing is compensated for in these constrained variants. From ***9*** onward, all peptides contained two or three helix-inducing constraints, with only the 25-mer **9** (52%), and 22-mers **10** (57%), **11** (63%), **20** (51%) and **24** (69%) displaying about twice the helicity of peptides **5**–**8** (30–35%).

### Peptides 12–17

Based on the above findings, six constrained peptide variants were synthesised: ***12–17***. ***12*** contained the three combined constraints of peptides ***9*** and ***10*** based on the scaffold of ***10*** and produced a comparable Tm of 52°C. ***13*** was the same as ***11*** but truncated by four residues from the C-terminus, and resulting in severely diminished binding and helicity. ***15*** was truncated by the same four residues as ***13*** with a similar bi-cyclic constraint; it was also unsuccessful and similarly displayed reduced helicity and poor thermal denaturation profile. ***16*** was identical to ***11*** with an ***a4*** changed from Ala to Phe. This was due to a hydrophobic pocket in the crystal structure that was anticipated to be occupied by the exchange, but this design was also unsuccessful. ***17*** was also based on ***11*** with the ***d3*** Leu changed to Cyclohexylalanine (Cha) as an alternative attempt to add hydrophobic bulk to the core in this central region. In particular, we wanted to increase the hydrophobicity in this part of the leucine zipper, since both ***13*** and ***15*** had failed in being truncated by four Leu residues relative to ***11***. ***12*** and ***17*** both represent NΔ10 truncations relative to JunW_CANDI_ and maintains seven of ten ***a/d*** core residues and five of six electrostatic ***e/g*** interactions.

### Peptides 18–24

From the success of ***12*** and ***17*** in the previous cohort, six further peptides were synthesised; all contained the same *f2*→*c3* and *b4*→*f4* constraint. ***18*** contained four Cha sidechains – one at each ***d*** position (see Figure 1). ***19*** contained two Cha residues at the central two ***d*** positions of the same template, with ***20*** containing two Cha residues at the outermost ***d*** positions of the same template. The final three peptides were identical to ***11*** but contained point mutations; Ile→Asn at ***a3*** to provide a partner for ***a3*** Lys in cFos, Lys→Arg at ***e2*** to provide an enhanced electrostatic contact with a ***g’1 Glu*** in cFos, and a Lys→Leu at ***g3*** for a potentially enhanced hydrophobic effect with ***e’4*** Leu in cFos. These changes were made in ***21–23*** respectively. Of these six, only ***20*** and ***22*** gave profiles consistent with a strong interaction affinity for cFos. For ***20***, placement of bulky hydrophobic groups at the outermost ***d*** positions helps to stabilise the dimer, possibly by also helping to constrain the helix to its target and aid in maintaining overall helicity. For ***22*** enhancing this electrostatic interaction would be predicted to add around 0.3 kcal/mol of stability [53], but clearly makes a large difference since ***21*** has a much lower affinity. Lastly, peptide ***24*** was synthesized to incorporate all of the changes introduced into ***12***, ***20***, and ***22*** - the three shortest and most effective peptides studied. This, the most helical peptide, was fifteen residues shorter than the JunW_CANDI_ parent peptide, ten residues shorter from the N-terminus than peptide 1, and more helical than both (Figure 1). Indeed, 24 was three and a half times more helical than JunW_CANDI_ (69% vs 19%) based on CD spectra. Lastly, conventional substitutions were made, as well as substituting leucine with the bulkier unnatural amino acid cyclohexylalanine (Cha) in 17–20 and 24 to increase both hydrophobicity and potentially the core packing in this part of the coiled coil. Unnatural amino acids at the ends of a peptide can potentially confer proteolytic stability to degradation by carboxy- or amino- peptidases. The CD data for peptides 17–24 suggest that placement of Cha groups at the temini enhances helicity. The two triply bridged peptides **12** (43%) and **24** (69%) differed only in the N-terminal Leu-Lys and C-terminal Leu in **12** being replaced by Cha-Arg and Cha respectively in **24**. The Cha residues contribute to this helix increase (cf. **20**, 51% vs. **12**, 43%) but there is an even larger helix induction through the Lys to Arg change (**20** vs **24**).

### Binding Affinity of Helix-constrained Peptides for cFos

Having identified a number of peptides able to adopt highly helical conformations upon introduction of the constraint, we sought to measure their binding affinities for cFos and explore relationships between affinity and helicity. Peptides **1**, **2**, **8**, **9**, **10**, **11**, **12**, **17**, **20**, **22**, and ***2*** **4** are compared in Figure 4 for their capacity to interact with cFos based on thermal denaturation experiments. Although there was no quantitative linear correlation between peptide helicity and affinity for cFos, there was a qualitative relationship. No peptides with <30% helicity were able to form a stable interaction with cFos and most peptides with high helicity also had high affinity for cFos, the mean helicity of interacting peptides being 49% versus 32% for non-interacting peptides. Despite truncating 15 residues from JunW_CANDI_ (>40% of all residues) and 10 residues from **1** (>30% of the molecule), peptide **24** had the highest Tm of 58°C. Similarly, most of the more helical peptides (e.g. **1**, **2**, **10**, **11**, **12**, **20**, **22**) had a Tm around 50°C, resulting in K_D_ values of ∼1–11 µM for binding to cFos (Table 1).

In addition to thermal denaturation profiles, isothermal calorimetric data could be obtained for all but one of the peptides in complex with cFos. To verify interactions between constrained peptides and cFos, we compared spectra for cFos alone, constrained peptide alone, and for the mixture of the two (Figure S1). For certain cFos-constrained peptide mixtures (e.g. <30% helicity), the observed spectra matched the average of the previous two spectra, indicating that no exchange of dimer had occurred. For some mixtures of cFos with a constrained peptide, the observed signal from the spectra exceeded that of the averaged homodimeric spectra, indicating binding. All peptides that perfomed well in thermal denaturation studies generated spectra that exceeded the average of component homodimers.

### Specificity of Helix-constrained Peptides for cFos

To establish if the truncated and constrained peptides retained the interaction specificity of the JunW_CANDI_ parent peptide, peptides capable of interacting with cFos were also incubated at equimolar concentrations with cJun and monitored via CD spectra. In addition, thermal denaturation was used to establish if an interaction took place, and therefore if the specificity exhibited by the JunW_CANDI_ parent was retained for constrained and truncated sequences. None of the peptides capable of forming an interaction with cFos were found to interact with cJun in these experiments (Figure S2, S3, S4), indicating specificity of the truncated helix constrained, peptides. In addition, a dimer exchange experiment was performed for the most helical peptide ***24.*** Spectra for a cJun-peptide solution and a cFos solution should, upon mixing, generate an averaged spectrum if there was no dimer exchange. However, helical spectra exceeded the average of the two component spectra, indicating that dimer exchange did occur. In contrast, for a cFos-peptide mixed with cJun, the average of the two component spectra was observed, indicating that no change in binding partner for peptide had occurred. Spectra for both mixtures can be superimposed (Figure S4).

### Thermodynamic Parameters for cFos Binding

**Figure 5:**
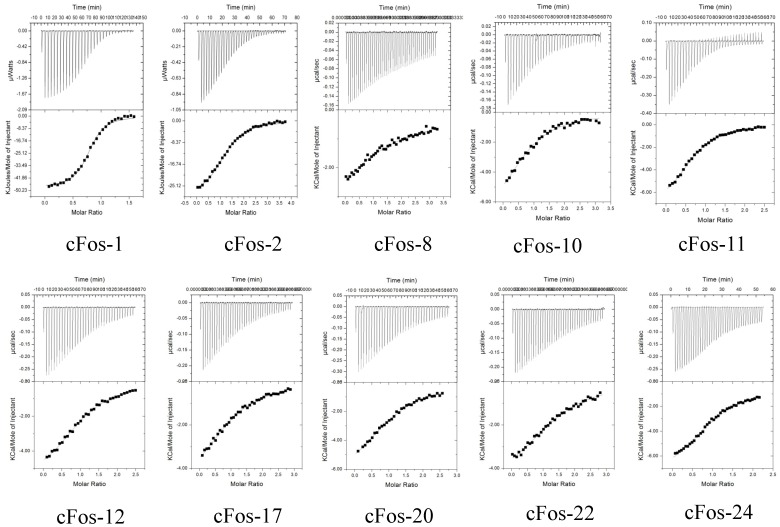
Isothermal Titration Calorimetry (ITC) analysis of leucine zipper domain interactions between constrained peptides and cFos. Shown are isotherms for all ten measureable heterodimers (1, 2, 8, 10, 11, 12, 17, 20, 22, and 24) injected into a cell containinginto cFos. The top and bottom panels show, respectively, raw data after baseline correction. During ITC experiments, approximately 200–600 µM of peptide A was injected in 30–40×5 µl batches from the injection syringe into the cell, which contained 10–40 µM cFos. Both partners were in a 10 mM Potassium Phosphate buffer, 100 mM Potassium Fluoride at pH 7. Experiments were undertaken at 20°C. The solid lines represent the fit of the data to the function based on the binding of a ligand to a macromolecule using the Microcal (GE Healthcare) Origin software [57].

To provide more insight to the origin of the binding affinity (K_D_) between helix-constrained peptides and cFos, we decided to dissect out the relative contributions of enthalpy versus entropy to the affinities. Isothermal Titration Calorimetry (ITC) experiments were conducted, enabling the free energy of binding to be split into entropic and enthalpic components (Figure 5 and Table 1), while also providing a stoichiometric measure of binding. Owing to the relative stabilities of the interacting pairs, only complexes formed between cFos and peptide ***1*** **,** ***2*** **,** ***8, 10*** **,** ***11*** **,** ***12*** **,** ***17*** **,** ***20*** and ***22*** and ***24*** were able to be characterised (see Table 1), with weaker associations formed by ***9*** unable to be assessed. For these and other pairs the thermodynamic parameters were unable to be determined by this technique owing to the large quantities of peptide required in both the cell and the syringe.

Thermodynamic parameters determined from ITC measurements on the above peptides indicate that the free energy of binding is predominantly driven by a favourable enthalpic term (ΔH −5.0 to −11.6 kcal/mol) with the entropic component either favourable or weakly opposing (Table 1). The free energy of binding for the longer parental JunW_CANDI_ peptide is driven by an even stronger enthalpic component (ΔH = −14.8 kcal/mol) but retarded by an unfavourable entropy term (TΔS = −6.9 kcal/mol). This is precisely as expected from the increased conformational entropy in the denatured state, relative to the more ordered helix-constrained peptides, that is lost upon binding to cFos [8]. Thus, truncation of the peptides has significantly reduced the enthalpic contribution to ΔG, but this is in part compensated for by a loss in the entropic barrier due to the helix-constraints. This is consistent with the helix-constraints pre-organizing the peptides into the receptor-binding conformation.

### Structure and Stability of 24

2D proton NMR spectra were obtained for peptide **24**. The NOE summary diagram (Figure 2) reveals small ^3^J_NHHα_ coupling constants, low temperature coefficients Δδ/T, and many d_αN(i, i+3)_ NOEs which collectively indicate that peptide **24** is heavily populated with helical structures in water. Strong *d*_αN(i,i+4)_ and *d*_αN(i,i+3)_, but only a few *d*_αβ(i,i+2)_, NOEs reveal an alpha-, rather than 3_10_-, helical structure. A structure calculation using these NMR restraints produced a highly convergent set of 20 lowest energy structures (RMSD = 1.87 Å over backbone heavy atoms) without any violations in distance restraints (>0.2 Å) or dihedral angles (>5°). The NMR-derived structures for peptide **24** are clearly in an alpha helical conformation (Figure 2), with an averaged backbone RMSD deviation from an idealized alpha helix of only 1.27 Å. However, the family of structures are somewhat flexible at the N- and C-terminal Cha residues. Figure 2 shows that thee three i→i+4 helix constraints are located on a helix face away from the face exposing the hydrophobic side chains (Cha1, I5, L8, A12, L15, I19, Cha22) for interaction with Fos peptide in a leucine zipper. This is consistent with the design intent to rigidly constrain the hydrophobic residues at desired positions of the heptad repeat for optimal exposure to cFos. Peptide **24** was also fairly stable in human serum over 4 h at 37°C, whereas the same sequences lacking the constraint were undetectable after 4 hours due to proteolytic cleavage (Figure 3).

## Discussion

One, two, or three i→i+4 helix constraints have been introduced into truncated peptide derived from a 37 residue peptide sequence from cJun (JunW_CANDI_), itself a weak antagonist of cJun binding to cFos. The aim was to investigate whether (a) much shorter helix-constrained peptides could maintain high affinity binding to cFos by virtue of pre-organizing the sequence in a conformation ideal for interaction with cFos [42], (b) key side chains in the Jun-based peptide represented sequence “hot spots” which were responsible for most of the energetic contributions to binding with cFos, (c) enthalpy or entropy contributions were most influential for binding, and (d) the downsized, helix-constrained, peptides retained high specificity for cFos, competing with cJun.

### Helix Induction

The use of i→i+4 helix-inducing constraints was successful in producing highly alpha helical peptides, allowing truncation of the sequence from 37 to 22 residues (but no shorter) without loss of helicity. A constraint near the C-terminus of the peptides (heptads 3 and 4) was more effective in inducing helicity and enhancing binding to cFos than a constraint introduced near the N-terminus (heptads 1 and 2). It was found that although CΔ7 truncation preserved some of the binding properties (**8–9**: Tm 32–38°C), NΔ10 deletion peptides (**10–24**: T_m_ 44–52°C) were much improved over NΔ14 deletions (**5–7**)**,** suggesting a key role for residues ***d2-g2***. In addition to constraint insertion, other amino acid substitutions were made to enhance core hydrophobic and electrostatic contacts. The addition of non-natural Cha sidechains to core residues within NΔ10 bicyclic peptides resulted in stabilisation with cFos for replacement of the two outermost ***d*** sidechains, suggesting that these act to constrain the helix to its target while maintaining overall helicity. In arriving at ***24*** from JunW_CANDI_ over 40% of the molecule was removed while still increasing overall helicity from 19 to 69%.

### Stability and Specificity

**Figure 6:**
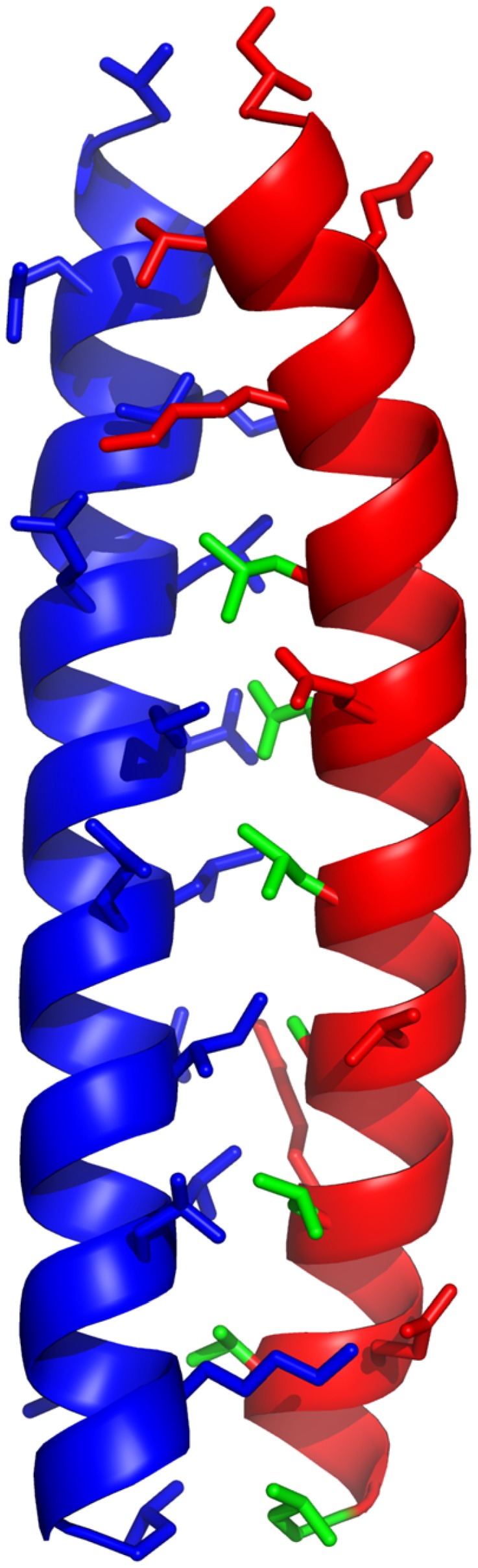
Coiled coil structure of the cJun-cFos interaction. Shown are the coiled coil regions of cJun (red) and cFos (blue) interaction (PDB coordinates: 1FOS [58]). Side-chains for interfacial **‘** ***a’ ‘d’ ‘e’ and ‘g’*** residues are shown and highlight the fact that the interactions between them are distributed broadly across the molecule. Key hydrophobic interfacial side-chains within cJun that are predicted to be required for effective binding to cFos are shown in green (***d2***, ***a3***, ***d3***, ***a4***, ***d4***, ***a5***, ***d5***, top to bottom: L, N, L, A, L, V, L).

While constraining heptads 3 or 4 at the C-terminus was most effective in promoting binding to cFos, truncating both heptads 1 and 2 did impact the binding suggesting that at least part of the second heptad from the N-terminus is required for effective binding. Subsequent designs incorporated residues ***d2-g2***, since the hydrophobic core appeared to be compromised if residue ***d2*** was missing, and thus binding affinity is broadly distributed along the length of the peptide from residues ***d2-d5*** (Figure 6). Qualitatively similar affinities were observed for all helix-constrained peptides, regardless of the size of the molecule. Importantly, all peptides capable of binding to cFos also retained specificity in the presence of cJun that was displayed by the CANDI-PCA selected parent peptide [42]. This was shown by dimer exchange experiments and thermal melt comparisons of equimolar solutions of peptide-cFos or peptide-cJun (Figures S2, S3, S4). Finally, the most helical peptide by CD measurements was **24**, which was examined by 2D-NMR spectroscopy and an alpha helical structure was verified along its length (Figure 2). This peptide was much more stable in human serum at 37° than the same peptide lacking the constraints, due to a consequence of the helix constraints and unnatural amino acids at each end (Figure 3). Degradative proteolytic enzymes in serum are known to recognize only denatured or extended strand peptide conformations in their active sites, with helical structures being too large and protective of peptide bonds to be cleaved by proteases [54], [55]. In addition to serum stability hydrocarbon restraints have additionally been speculated to facilitate cell membrane permeability, with several studies demonstrating this directly [7], [15].

### Thermodynamics

Consistent with a reduction in conformational freedom imposed by the helix constraints, there is a measurable increase in the entropic contribution to cFos binding. TΔS was −6.9 kcal/mol for cFos-JunW_CANDI_ versus −3.8 to +1.6 kcal/mol for a cFos-constrained peptide pair, with the triply bridged helical peptide **12** exhibiting a 7.5 kcal/mol more positive TΔS than for JunW_CANDI_. This contrasts with the weaker binding, but more helical, **24** where the Cha-Arg at the N-terminus and Cha at the C-terminus appear to disfavour binding. Thus favourable free energy changes on binding to cFos stem from a combination of enthalpic contributions, reduced upon peptide truncation, and more importantly, increased conformational entropy that arises from introduction of the helix-inducing constraints. This contrasts with the cFos-JunW_CANDI_ interaction which was driven primarily by a larger enthalpy than for the much shorter constrained peptides. Because the coiled coil requires interactions along its entire length for thermodynamic stability, truncation of the peptide causes a large enough loss in enthalpy to compensate for the conformational entropy advantage that is conferred by the helix constraints. Thus the extent of sequence truncation has been limited here to splicing ∼40% off the sequence and this balancing act, between focussed entropic targeting of hot spots in a coiled coil (such as helical heptads at the C-terminus in this case) and losing enthalpy due to removal of many of the interactions that stabilize the coiled coil [56], may be more difficult to achieve than for other helical protein surfaces where there are more pronounced hot spots. In particular for ***24***, hydrophobic residues ***d2, a3, d3, a4, d4, a5*** and ***d5*** (Cha, I, L, A L, I, Cha) are predicted to make large enthalpic contributions to coiled-coil binding potency.

In summary, the affinity of cJun for cFos is dependent upon interactions along the entire length of these two helical coils, with the two C-terminal heptads contributing a little more enthalpy to the interaction energy than the two N-terminal heptads. Thus, shortening the sequence by over 40% from JunW_CANDI_ to peptide 24 has correspondingly and proportionately reduced the free energy and enthalpy of interaction, which has been compensated for to some extent by pre-organizing the peptide in a stable alpha helical conformation. This has led to peptides that bind to cFos in the µM affinity range. In the future it may be possible to retain or further improve the affinity of peptide interactions with cFos by using further iterations of truncating the sequence, with alternative positioning of helix-conferring constraints, such as these or others, combined with non-natural amino acids, HBS approaches or the use of β-peptides to generate even smaller peptidomimetics of AP-1 components. However, the large surface area of interaction, the shallow binding pockets, and especially the coiled coil nature of the heterodimer make this and other transcription factors more challenging than many other protein alpha helices to mimic using smaller helix-constrained peptides. The extent to which helix-constrained transcription factors can be shortened is uncertain but this does represent a promising approach to generating smaller transcriptional regulators. This research is particularly timely given the rapid increase in knowledge from proteomics and interactomics studies on transcriptional regulators in signalling pathways.

## Supporting Information

Figure S1 **CD spectra for all constraints in this study.** These are shown both in isolation and as a mixture with cFos. Data have been collected by measuring the level of helicity at 222 nm in an applied photophysics chirascan Circular Dichroism (CD) Spectrometer. Data have been converted from raw ellipticity to Molar Residue Ellipticity (MRE). From these raw data it is possible to see which heterodimers constitute an increase over the average of the homodimeric components (black dotted line). Any increase in the helical signal that exceeds the average of cFos (black) and the constrained peptide (blue), that would be anticipated for a non-interacting pair, is clearly observed in the heterodimeric profiles (red) and therefore strongly indicates the presence of an interaction.
(TIFF)

Figure S2 **Raw thermal melting data for homo and heterodimeric complexes with cJun for constrained peptides** ***1*** **,** ***2*** **,** ***8*** **,** ***9, 10*** **,** ***11, 12, 17, 20, 22, and 24*** **.** Shown are raw thermal melting data for all homo and heterodimeric complexes. Data have been collected by measuring the level of helicity at 222 nm in an applied photophysics chirascan Circular Dichroism (CD) Spectrometer. Data have been converted from raw ellipticity to Molar Residue Ellipticity (MRE) according to equation 1 to take account of the different peptide lengths. Thermal melting data for cJun is shown in black, data for the constrained peptide in isolation is shown in blue, the average of these two as a black dotted line, with the cJun/constrained peptide mixture is shown in red. It is clear that none of the peptides form a stable interaction with cJun.
(TIFF)

Figure S3 **CD spectra as MRE both in isolation and as a mixture with cJun.** From these raw data it is also clear that no interaction is occurring between constrained peptides and cJun. Rather, specta appear as averages of their homodimeric components (i.e. superimpose with the homomeric averages). We observe no heteromeric helical signal (red) that exceeds the average (black dotted line) of cJun (black) and the constrained peptide (blue), that would be anticipated for a non-interacting pair.
(TIFF)

Figure S4 **Dimer exchange experiments between cJun, cFos and constrained peptide** ***24*** **.** **a)** Equimolar mixures of cJun-cJun and cFos-24 are mixed and the observed signal closely resembles the average of the two constituent spectra, indicating no change has occurred. **b)** Equimolar mixtures of cFos-cFos and cJun-24 are mixed and the observed spectra greatly exceeds the average of the two constituent spectra, indicating that dimer exchange has occurred. **c)** Mixtures from a) and b) superimpose, indicating that the same species is populated in both cases.
(TIFF)

Table S1 **Helical Data obtained via Circular Dichroism.** Shown are A) homo and B) heteromeric samples. Column 1 displays the 222/208 ratio which can be used as an indication of the presence of coiled coils. A ratio higher than 1 is generally indicated evidence that a coiled coil has formed, while a ratio of less than 0.9 is taken to indicate the presence of isolated helices. Shown in column two are the calculated fractional helicities taken from the Molar Residue Ellipticity at 22 nm. Fraction helicity (ƒH) can be calculated as ƒH = (θ_222_−θ_c_)/(θ_222∞_−θ_c_) where θ_222∞_ = (−44000+250*T)*(1−k/Nr) and θ_c_ = 2220–(53*T). In these equations the wavelength dependent constant k = 2.4 (at 222 nm), Nr = the number of residues, and T = 20 degrees Celsius (293 K). The 222/208 ratio is used to provide evidence on whether the helices are monomeric or are adopting a quaternary structure. Measured helicity for the original JunW_CANDI_ peptide and for the template on which the hydrocarbon constraints have been introduced. These data take into account the constrained variants which have been shown to introduce significant helicity into the molecule. The N- and C-capping motifs have been removed for the constrained since they are considered to be largely redundant upon their introduction.
(DOC)

Table S2 **Assigned ^1^H NMR signals for peptide 24 in H_2_O:D_2_O (9∶1) at 298** **K.**
(DOC)

File S1 **Supporting Information.**
(DOC)
