# Supplementary figures and images for: Truncated and Helix-Constrained Peptides with High Affinity and Specificity for the cFos Coiled-Coil of AP-1
Source: PLoS One. 2013 Mar 27;8(3):e59415. doi: 10.1371/journal.pone.0059415 (PMC3609778; doi:10.1371/journal.pone.0059415)

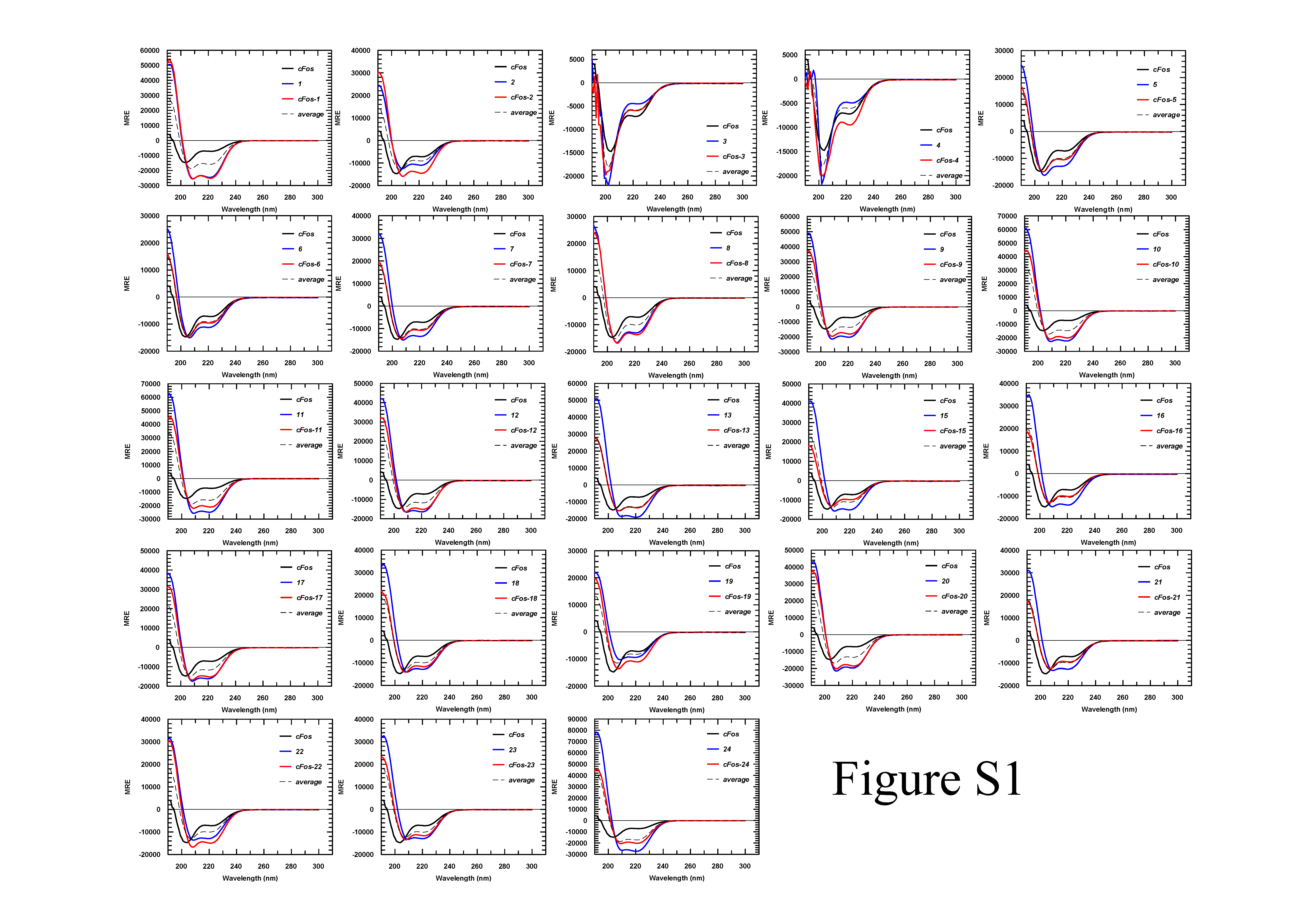

Supplement: Figure S1 — CD spectra for all constraints in this study. These are shown both in isolation and as a mixture with cFos. Data have been collected by measuring the level of helicity at 222 nm in an applied photophysics chirascan Circular Dichroism (CD) Spectrometer. Data have been converted from raw ellipticity to Molar Residue Ellipticity (MRE). From these raw data it is possible to see which heterodimers constitute an increase over the average of the homodimeric components (black dotted line). Any increase in the helical signal that exceeds the average of cFos (black) and the constrained peptide (blue), that would be anticipated for a non-interacting pair, is clearly observed in the heterodimeric profiles (red) and therefore strongly indicates the presence of an interaction. (TIFF) [file pone.0059415.s001.tiff]

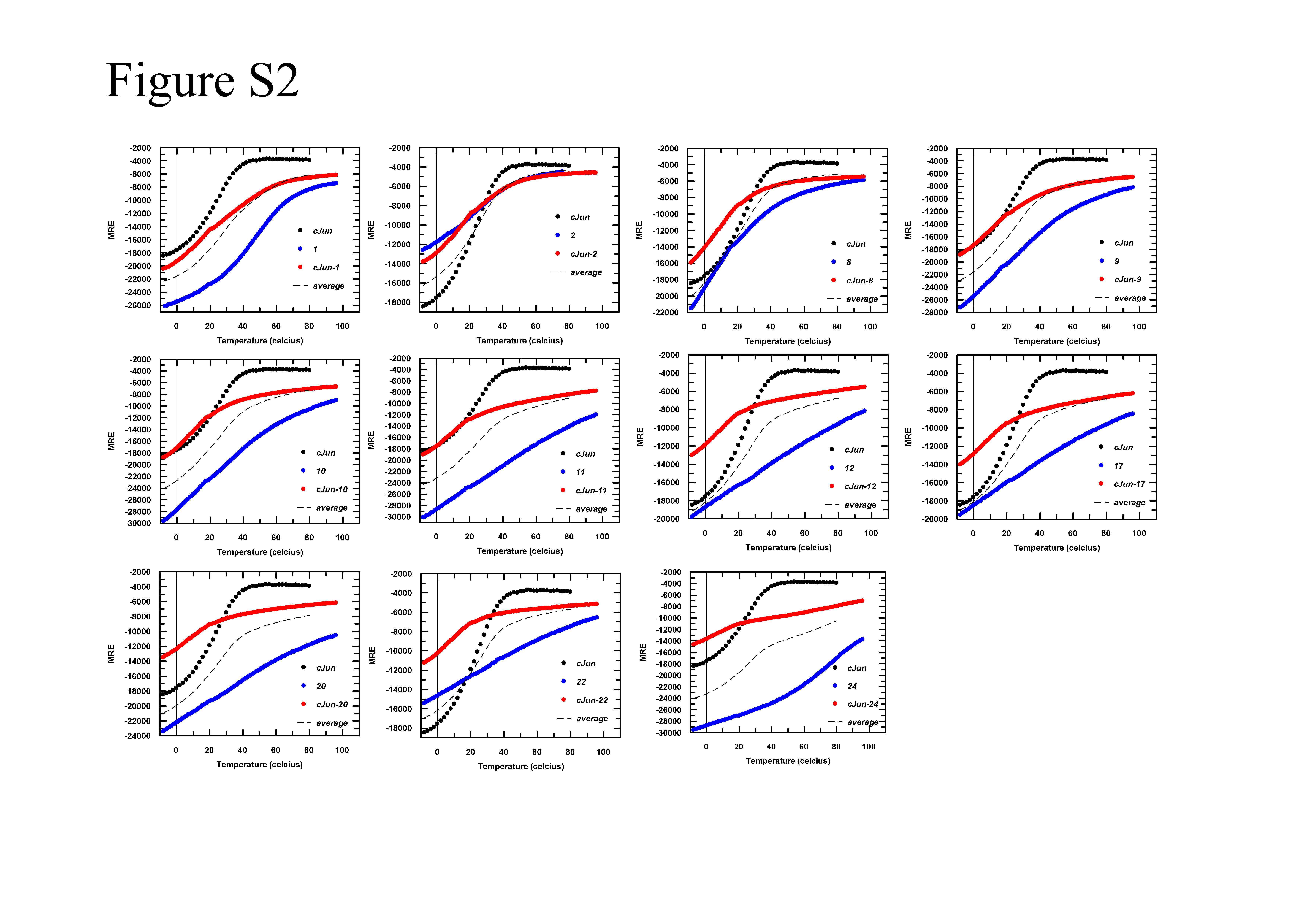

Supplement: Figure S2 — Raw thermal melting data for homo and heterodimeric complexes with cJun for constrained peptides 1 , 2 , 8 , 9, 10 , 11, 12, 17, 20, 22, and 24 . Shown are raw thermal melting data for all homo and heterodimeric complexes. Data have been collected by measuring the level of helicity at 222 nm in an applied photophysics chirascan Circular Dichroism (CD) Spectrometer. Data have been converted from raw ellipticity to Molar Residue Ellipticity (MRE) according to equation 1 to take account of the different peptide lengths. Thermal melting data for cJun is shown in black, data for the constrained peptide in isolation is shown in blue, the average of these two as a black dotted line, with the cJun/constrained peptide mixture is shown in red. It is clear that none of the peptides form a stable interaction with cJun. (TIFF) [file pone.0059415.s002.tiff]

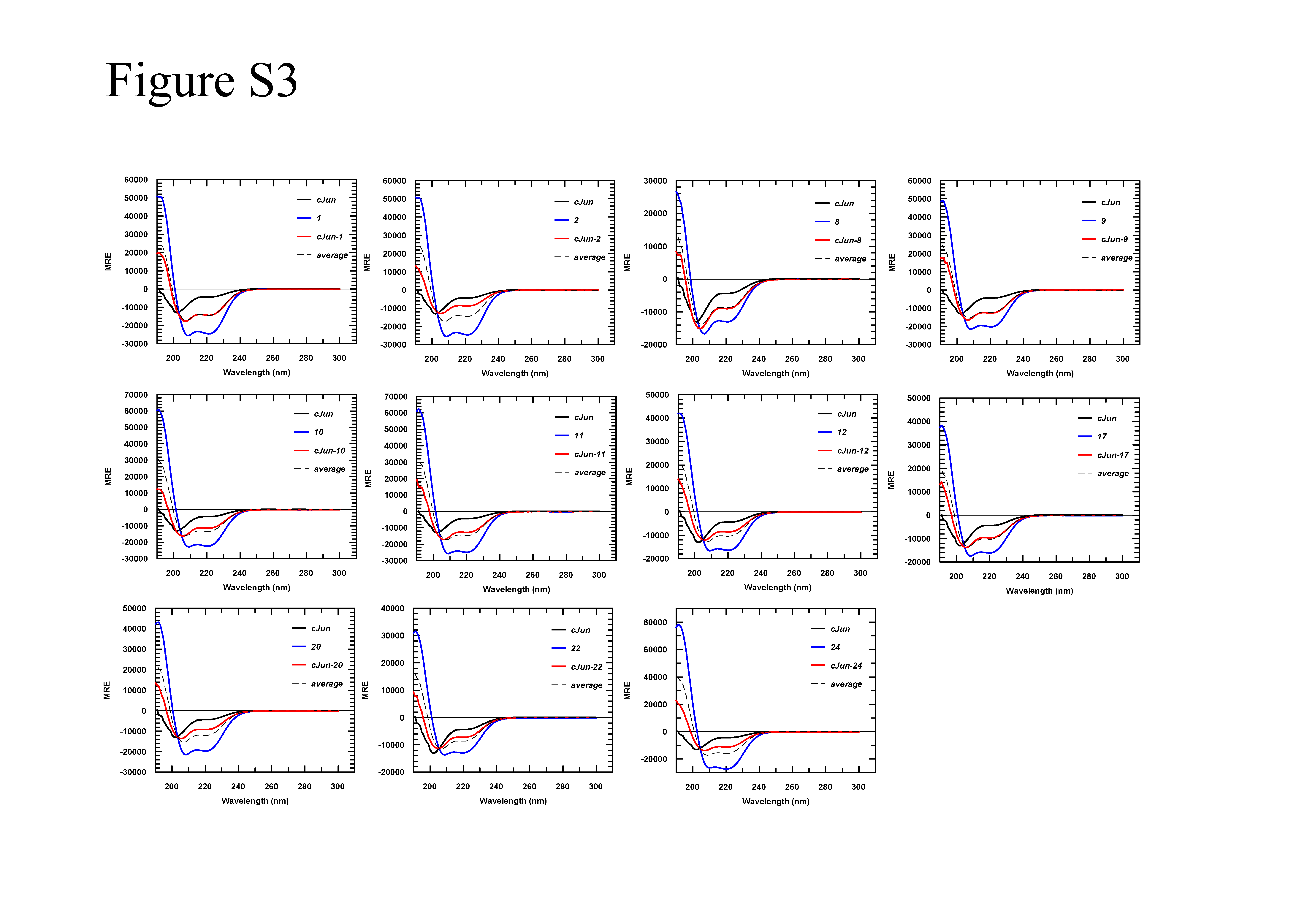

Supplement: Figure S3 — CD spectra as MRE both in isolation and as a mixture with cJun. From these raw data it is also clear that no interaction is occurring between constrained peptides and cJun. Rather, specta appear as averages of their homodimeric components (i.e. superimpose with the homomeric averages). We observe no heteromeric helical signal (red) that exceeds the average (black dotted line) of cJun (black) and the constrained peptide (blue), that would be anticipated for a non-interacting pair. (TIFF) [file pone.0059415.s003.tiff]

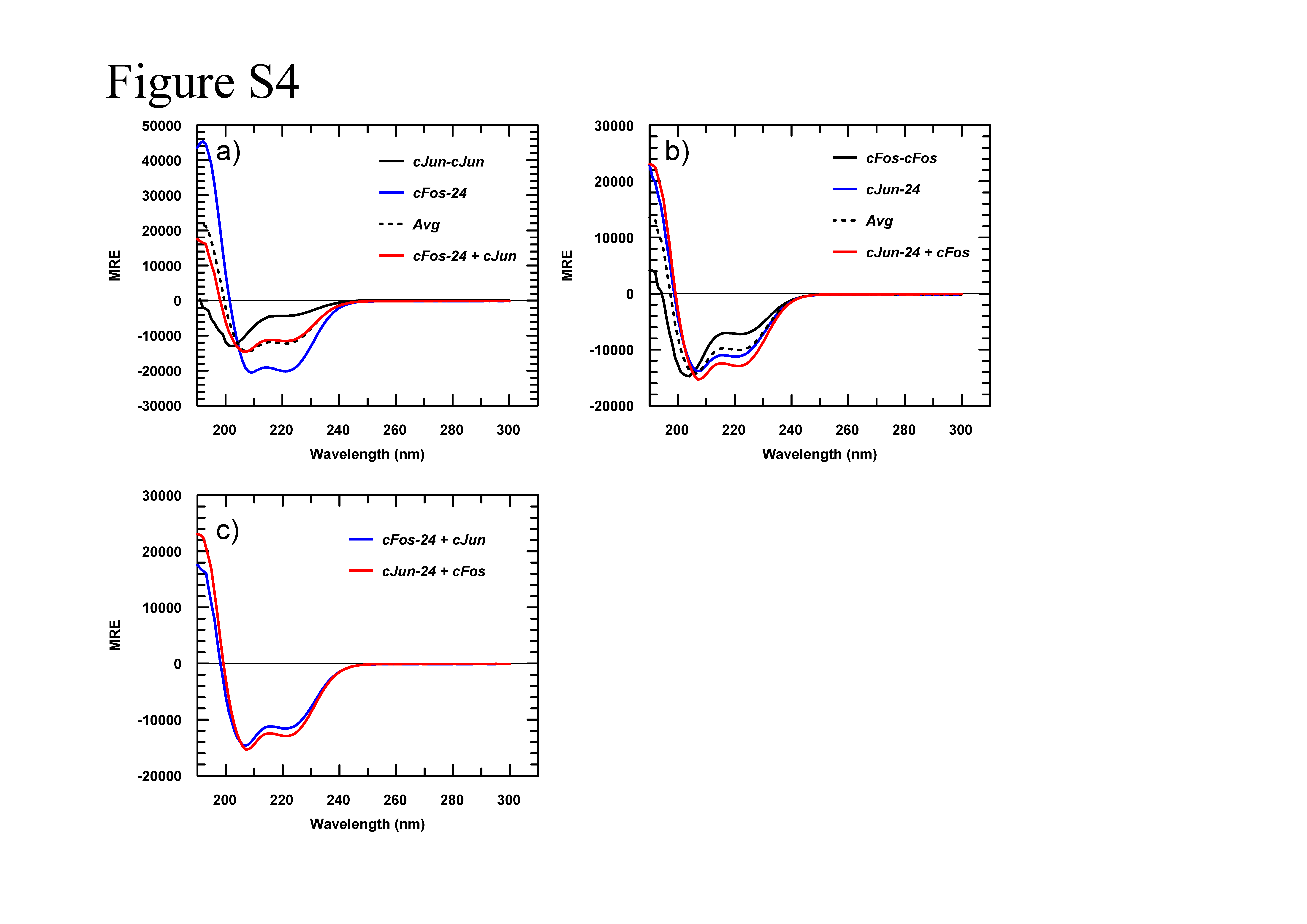

Supplement: Figure S4 — Dimer exchange experiments between cJun, cFos and constrained peptide 24 . a) Equimolar mixures of cJun-cJun and cFos-24 are mixed and the observed signal closely resembles the average of the two constituent spectra, indicating no change has occurred. b) Equimolar mixtures of cFos-cFos and cJun-24 are mixed and the observed spectra greatly exceeds the average of the two constituent spectra, indicating that dimer exchange has occurred. c) Mixtures from a) and b) superimpose, indicating that the same species is populated in both cases. (TIFF) [file pone.0059415.s004.tiff]
