# Supplementary material for: Truncated and Helix-Constrained Peptides with High Affinity and Specificity for the cFos Coiled-Coil of AP-1
Source: PLoS One. 2013 Mar 27;8(3):e59415. doi: 10.1371/journal.pone.0059415 (PMC3609778; doi:10.1371/journal.pone.0059415)
Supplement: Table S1 — Helical Data obtained via Circular Dichroism. Shown are A) homo and B) heteromeric samples. Column 1 displays the 222/208 ratio which can be used as an indication of the presence of coiled coils. A ratio higher than 1 is generally indicated evidence that a coiled coil has formed, while a ratio of less than 0.9 is taken to indicate the presence of isolated helices. Shown in column two are the calculated fractional helicities taken from the Molar Residue Ellipticity at 22 nm. Fraction helicity (ƒH) can be calculated as ƒH = (θ222−θc)/(θ222∞−θc) where θ222∞ = (−44000+250*T)*(1−k/Nr) and θc = 2220–(53*T). In these equations the wavelength dependent constant k = 2.4 (at 222 nm), Nr = the number of residues, and T = 20 degrees Celsius (293 K). The 222/208 ratio is used to provide evidence on whether the helices are monomeric or are adopting a quaternary structure. Measured helicity for the original JunWCANDI peptide and for the template on which the hydrocarbon constraints have been introduced. These data take into account the constrained variants which have been shown to introduce significant helicity into the molecule. The N- and C-capping motifs have been removed for the constrained since they are considered to be largely redundant upon their introduction. (DOC) [file pone.0059415.s005.doc]

|  | **Homotypic**  **Ɵ_222_/_208_**  **(20ºC)** | **Fraction helical (ƒH)** | **Peptide-cFos**  **Ɵ_222_/_208_**  **(20ºC)** | **Peptide-cFos**  **Fraction helical (ƒH)** | **Peptide-cJun**  **Ɵ_222_/_208_**  **(20ºC)** | **Peptide-cJun**  **Fraction helical (ƒH)** |
| --- | --- | --- | --- | --- | --- | --- |
| **cFos** | 0.59 | 0.20 | -- | -- | -- | -- |
| **JunW_CANDI_** | 0.79 | 0.19 | 0.97 | 0.42 | -- | -- |
| **1** | 0.96 | 0.63 | 1.00 | 0.65 | 0.82 | 0.38 |
| **2** | 0.87 | 0.30 | 0.91 | 0.39 | 0.73 | 0.24 |
| **3** | 0.37 | 0.14 | 0.48 | 0.17 | -- | -- |
| **4** | 0.36 | 0.15 | 0.61 | 0.26 | -- | -- |
| **5** | 0.78 | 0.34 | 0.73 | 0.28 | -- | -- |
| **6** | 0.76 | 0.30 | 0.71 | 0.26 | -- | -- |
| **7** | 0.88 | 0.35 | 0.76 | 0.29 | -- | -- |
| **8** | 0.79 | 0.34 | 0.84 | 0.38 | 0.67 | 0.25 |
| **9** | 0.92 | 0.52 | 0.92 | 0.47 | 0.78 | 0.34 |
| **10** | 0.99 | 0.57 | 0.96 | 0.52 | 0.74 | 0.31 |
| **11** | 0.96 | 0.63 | 0.96 | 0.55 | 0.76 | 0.34 |
| **12** | 0.99 | 0.43 | 0.92 | 0.40 | 0.74 | 0.24 |
| **13** | 1.04 | 0.49 | 0.88 | 0.36 | -- | -- |
| **15** | 0.95 | 0.39 | 0.76 | 0.27 | -- | -- |
| **16** | 0.95 | 0.37 | 0.80 | 0.28 | -- | -- |
| **17** | 0.91 | 0.42 | 0.90 | 0.40 | 0.73 | 0.26 |
| **18** | 0.91 | 0.34 | 0.83 | 0.32 | -- | -- |
| **19** | 0.90 | 0.26 | 0.81 | 0.30 | -- | -- |
| **20** | 0.91 | 0.51 | 0.92 | 0.49 | 0.71 | 0.26 |
| **21** | 0.94 | 0.33 | 0.78 | 0.26 | -- | -- |
| **22** | 0.94 | 0.34 | 0.89 | 0.40 | 0.69 | 0.21 |
| **23** | 0.99 | 0.34 | 0.87 | 0.32 | -- | -- |
| **24** | 1.08 | 0.69 | 1.00 | 0.52 | 0.81 | 0.30 |
