# Supplementary material for: Truncated and Helix-Constrained Peptides with High Affinity and Specificity for the cFos Coiled-Coil of AP-1
Source: PLoS One. 2013 Mar 27;8(3):e59415. doi: 10.1371/journal.pone.0059415 (PMC3609778; doi:10.1371/journal.pone.0059415)
Supplement: Table S2 — Assigned 1H NMR signals for peptide 24 in H2O:D2O (9∶1) at 298 K. (DOC) [file pone.0059415.s006.doc]

| **Residue** | NH (ppm) | Hα (ppm) | Hβ (ppm) | Others (ppm) |
| --- | --- | --- | --- | --- |
| Ac-Cha (1) | 8.18 | 4.12 | 1.59 | Hγ 1.50; Hδ 1.33; Hε 1.04; HZ 1.34  Ac(CH_3_) 1.97 |
| R (2) | 8.21 | 4.01 | 1.74 | Hγ 1.64; Hδ 3.10; Hε 7.13 |
| K (3) | 8.01 | 4.11 | 1.53 | Hγ 1.30; Hδ 1.69; Hε 3.60; Hz 8.10 |
| E (4) | 8.20 | 4.01 | 1.74 | Hγ 2.48 |
| I (5) | 7.74 | 3.71 | 1.86 | Hγ 1.52, 1.17; Hδ 0.84 |
| Y (6) | 8.10 | 3.91 | 2.98 | Hδ 7.07; Hε 7.07 |
| D (7) | 9.07 | 4.48 | 2.71, 2.97 |  |
| L (8) | 7.91 | 4.08 | 1.85 | Hγ 1.52; Hδ 0.81 |
| R (9) | 7.77 | 3.98 | 1.73 | Hγ 1.65; Hδ 3.03; Hε 7.09 |
| K (10) | 7.78 | 3.90 | 1.83 | Hγ 1.47; Hδ 1.62; Hε 3.62; Hz 8.15 |
| K (11) | 7.84 | 4.04 | 1.74 | Hγ 1.47; Hδ 1.60; Hε 3.58 |
| A (12) | 7.72 | 4.05 | 1.39 |  |
| N (13) | 8.49 | 4.33 | 2.76, 2.70 | Hδ 7.55, 6.73 |
| D (14) | 9.07 | 4.55 | 2.98, 2.71 |  |
| L (15) | 7.92 | 4.25 | 1.73 | Hγ 1.67;  Hδ 0.84 |
| R (16) | 7.66 | 3.89 | 1.89 | Hγ 1.73; Hδ 3.16; Hε 7.19 |
| K (17) | 7.73 | 4.18 | 1.84 | Hγ 1.53; Hδ 1.73; Hε 3.72; Hz 8.00 |
| H (18) | 7.94 | 4.17 | 3.51, 3.30 | Hδ 7.10; Hε 8.60 |
| I (19) | 7.80 | 3.63 | 1.79 | Hγ1 1.69, 1.17; Hγ2 0.83; Hδ 0.83 |
| A (20) | 8.21 | 4.01 | 1.36 |  |
| D (21) | 8.12 | 4.61 | 2.89, 2.63 |  |
| Cha (22) | 7.27 | 4.04 | 1.67 | Hγ 1.41; Hδ 1.52; Hε 0.78; HZ 1.06 |
| NHT | 6.73, 7.04 | -- | -- | -- |
