# Supplementary material for: Truncated and Helix-Constrained Peptides with High Affinity and Specificity for the cFos Coiled-Coil of AP-1
Source: PLoS One. 2013 Mar 27;8(3):e59415. doi: 10.1371/journal.pone.0059415 (PMC3609778; doi:10.1371/journal.pone.0059415)
Supplement: File S1 — Supporting Information. (DOC) [file pone.0059415.s007.doc]

**Supporting Information.**

***ITC Measurements -***ITC measurements were made using a Microcal VP-ITC instrument with data collected and processed using the Origin 7.0 software package. All measurements were carried out at least two times. Briefly, all peptides were studied at 20°C in 10 mM potassium phosphate and 100 mM potassium fluoride at pH 7. 600 μL of peptide 1 was loaded into the syringe at between 175 μM and 450 μM peptide concentration. 1800 μL of peptide **2** was loaded into the cell at 10-40 μM. The peptide in the syringe and cell were reversed to check that the results were unaffected by this change. The experiment was undertaken by injecting 5 μL x 35 injections of peptide **1** into the calorimetric cell. The change in thermal power as a function of each injection was automatically recorded using the Microcal Origin software and the raw data were integrated to yield ITC isotherms of heat release per injection as a function of Fos to Jun Molar ratio (Figure 5). In general the concentration of peptide **2** loaded into the cell was thirty times the anticipated PPI K_D_ and the concentration of peptide **1** in the syringe was at least twenty times the concentration of peptide **2**. No precipitation of protein was observed in any of the experiments undertaken. Following ITC measurements, the data were fit to a one-site model:

$q\left(i\right)=\left(\left(n\Delta HVP\right)/2\right)\left[1+\left(L/nP\right)+\left(Kd/nP\right)\right]-\left[\left[1+\left(L/nP\right)+\left(Kd/nP\right)\right]^{2}-\left(4L/nP\right)\right]^{1/2}$ [3]

where q(i) is the heat release (kcal/mol) for the *i*th injection, n is the stoichiometry of heterodimerisation, V is the effective volume of protein sample loaded into the calorimetric cell (1.46 mL), P is the total Jun concentration in the calorimetric cell (μM) and L is the total Fos concentration in the calorimetric cell at the end of each injection (μM). This model is derived from the binding of a ligand to a macromolecule using the law of mass action (assuming a 1:1 stoichiometry) to extract the various thermodynamic parameters , namely the apparent equilibrium constant (Kd) and the enthalpy change (ΔH) associated with heterodimerisation. The free energy change (ΔG_bind_) upon ligand binding can be calculated from the relationship:

$\Delta G_{bind}=-RTLnK_{D}$ [4]

where R is the universal molar gas constant (1.9872 cal·K^-1^·mol^-1^), T is the absolute temperature in Kelvin (293.15 K) and K_D_ is the dissociation constant of binding with units of mol·L^-1^. Finally, the entropic contribution (TΔS) to the free energy of binding was calculated using the derived values of ΔH and ΔG_bind_.

**Supporting Information References**

1. Wiseman T, Williston S, Brandts JF, Lin LN (1989) Rapid measurement of binding constants and heats of binding using a new titration calorimeter. Anal Biochem 179: 131-137.

2. Seldeen KL, McDonald CB, Deegan BJ, Farooq A (2008) Coupling of folding and DNA-binding in the bZIP domains of Jun-Fos heterodimeric transcription factor. Arch Biochem Biophys 473: 48-60.

3. Worrall JA, Mason JM (2010) Thermodynamic analysis of Jun-Fos coiled coil peptide antagonists. FEBS J.
